# The Fate of the Jailed Branch: Challenging the Dogma of PDA Stenting in Cases with a Pulmonary Artery Branch Originating from the PDA

**DOI:** 10.1007/s00246-025-03874-w

**Published:** 2025-05-02

**Authors:** Mercy Ng’eno, Brent M. Gordon, Rohit Rao, Justin R. Ryan, Jessica Haley, Srujan Ganta, John Nigro, Howaida El-Said

**Affiliations:** 1 Department of Pediatrics, University of California San Diego, La Jolla, San Diego, CA USA; 2 Department of Cardiology, Rady Children’s Hospital Division of Cardiology, 3020 Children’s Way MC 5004, San Diego, CA 92123 USA; 3 Helen and Will Webster Foundation 3D Innovations Lab, Rady Children’s Hospital, San Diego, CA USA; 4 Department of Neurological Surgery, UC San Diego Health, La Jolla, San Diego, CA USA; 5 Department of Surgery, University of California San Diego, La Jolla, San Diego, CA USA

**Keywords:** Isolated branch pulmonary arteries, pulmonary artery coarctation, LPA stenosis, PDA stent complications

## Abstract

Patent ductus arteriosus (PDA) stenting is evolving as the preferred method for establishing stable pulmonary blood flow in cyanotic infants, offering lower mortality, faster recovery, and shorter hospital stays. However, stenting may lead to branch pulmonary artery (BPA) jailing, potentially restricting blood flow to the jailed branch. This study analyzes patient characteristics, cardiovascular anatomy, stenting techniques, and outcomes involving BPA jailing where the BPA originates from the PDA, with a focus on the growth of the jailed BPA. A retrospective review was conducted of infants with duct-dependent pulmonary blood flow who underwent PDA stenting at Rady Children’s Hospital San Diego from 2013 to 2024. Of 70 infants, 22 (31%) experienced BPA jailing due to the BPA originating from the PDA. The median age and weight at stenting were 9.5 days and 3275 g, with PDA as the sole source of pulmonary blood flow in 72% of cases. PDA type III was the most prevalent (77%). The jailed BPA showed significant distal growth (mean Nakata index increase of 117.35 mm^2^/m^2^, p = 0.0001), with symmetry maintained. Re-intervention for hypoxia was required in 55% of cases and involved ballooning, re-stenting, and strut dilation. There were no 30-day mortalities (2 late deaths occurred). Progression to Glenn palliation occurred in 12 patients, 5 had a definitive repair, 1 underwent left pulmonary artery plasty and a BT shunt and 2 await repair. PDA stenting in infants with a BPA originating from the PDA is feasible with notable distal BPA growth despite frequent re-interventions. Patients maintained pulmonary artery symmetry with excellent survival.

## Introduction

Infants born with duct-dependent pulmonary blood flow require either neonatal definitive repair or an intervention to establish pulmonary blood flow as a palliative interim procedure before further surgical management. The next step for patients who received palliation can be a definitive surgical repair or 2nd stage palliation in the setting of single ventricle patients. With the advancement in knowledge, techniques, and technology associated with patent ductus arteriosus (PDA) stenting, this procedure has become the preferred primary intervention to establish a stable source of pulmonary blood flow in cyanotic infants [1–4]. This preference is attributed to its comparable efficacy to systemic-pulmonary surgical shunts, lower mortality rate, better post-procedure recovery, and reduced hospital stay [1–9].

Branch pulmonary artery (BPA) jailing is a potential scenario in PDA stenting. It occurs when the stent extends beyond the PDA into the MPA or a BPA causing entrapment of one or both branches and can lead to restriction of blood flow. The incidence of BPA jailing is reported to be 22% [10]. In the early years of PDA stenting, BPA stenosis was considered an important risk factor for jailing and thus a contraindication to PDA stenting. The American Heart Association 2011 statement on *Indications for Cardiac Catheterization and Intervention in Pediatric Cardiac Disease* included a class III recommendation (should not be performed) against ductal stenting in infants with cyanotic congenital heart disease who have obvious proximal Pulmonary artery stenosis in the vicinity of the ductal insertion (level of evidence: C) [11]. This has however been challenged over the years with several authors suggesting that PDA stenting can be safely performed in patients with BPA stenosis without inferior outcomes [1, 12].

**Fig. 1:**
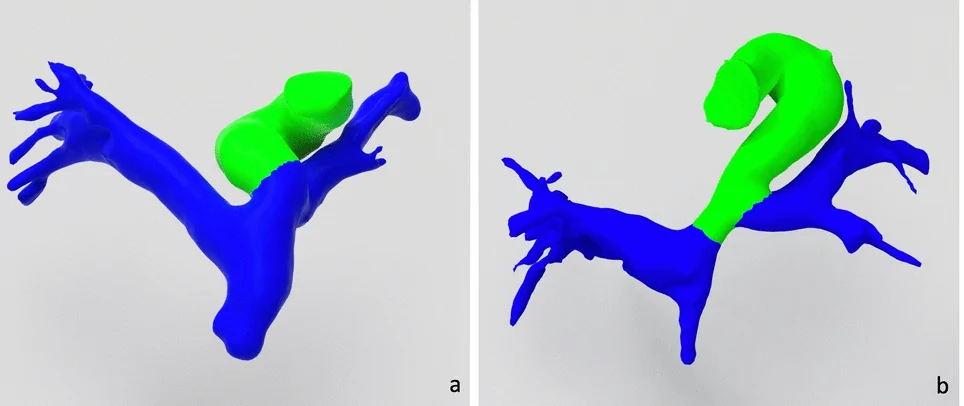
(**a**) Shows continuous branch pulmonary arteries with the PDA entering at the LPA/MPA junction, whereas (**b**) illustrates the LPA arising from the PDA with offsetting of the branch pulmonary arteries and PDA tissue maintaining continuity, BPAs are continuous and connected to the MPA. In (**a**), stenting of the PDA could inadvertently jail the LPA, while in (**b**), PDA stenting results in unavoidable jailing of the branch pulmonary arteries

A critical distinction however emerges between the BPA inadvertently jailed by the PDA stent and the BPA arising from the PDA (Fig. 1). While both scenarios involve crossing of the BPA, the latter involves a branch PA that is inevitably crossed and jailed by the stent.

The purpose of our study was to analyze patient characteristics, cardiovascular anatomy, PDA stenting techniques, and outcomes in patients with BPA jailing due to a BPA arising from the PDA. Our primary endpoint was the growth of the jailed pulmonary artery branch, as measured by the Nakata index and symmetry score. Secondary endpoints were complications, reintervention, interstage mortality, and other clinical outcomes.

## Methods

This study is a single-center retrospective chart review conducted at Rady Children’s Hospital and approved by the University of California, San Diego Institutional Review Board. Informed consent was waived and participants who met the following inclusion/exclusion criteria were included in the study.

### Inclusion Criteria

- All infants with duct-dependent pulmonary blood flow and a branch pulmonary artery arising from the PDA who underwent trans-catheter PDA stenting as the initial palliative procedure at Rady Children’s Hospital San Diego (RCHSD) cardiac catheterization lab between January 1, 2013, and July 31, 2024.

### Exclusion Criteria

- Patients with duct-dependent pulmonary blood flow who received surgical aortopulmonary shunts (modified Blalock-Taussig Thomas shunt) as the initial palliative procedure or those who underwent neonatal definitive repair.
- Infants with duct-dependent pulmonary blood flow who underwent trans-catheter PDA stenting as the initial palliative procedure but did not have a branch pulmonary artery arising from the PDA
- Infants with 1 or both PAs arising from a ductus arteriosus with discontinuity between contralateral BPA and/or MPA

In 2018 the institutional practice at RCHSD changed from selective PDA stenting for neonates with duct-dependent pulmonary blood flow to PDA stenting as an initial palliative procedure. PDA stenting is now pursued for all neonates with duct-dependent pulmonary blood flow who cannot undergo primary repair [14].

### Branch Pulmonary Artery Arising from the PDA

This was defined as a branch pulmonary artery arising from the PDA, with ductal tissue maintaining continuity with one or both branch pulmonary arteries. While the branch PAs remain continuous and connected to the MPA, they are at risk of discontinuity following PDA closure. This was identified as offsetting of the branch pulmonary arteries on angiogram or 3D reconstructed cardiac CT (Fig. 1). This is similar to what Elzenga et al. describes as an abnormal PDA connection, where the entry site of the PDA is displaced toward one of the branch pulmonary arteries. In their study, histological examination revealed ductal tissue within the walls of a pulmonary artery in more than half of the cases [15].

This differs from Unilateral Ductal Origin of Pulmonary Artery (DOPA), as described by other authors, where the affected PA receives blood solely from a PDA and has no connection to the MPA, while the contralateral PA is normally connected to the MPA. In cases of Bilateral DOPA, each PA arises from a separate ductus, with no continuity with the MPA [16, 17]. These variations are all part of the morphological spectrum of Ductal Origin of the Pulmonary Artery (DOPA), resulting from the persistent connection of the hilar pulmonary artery to the distal sixth aortic arch, which is destined to become the ductus arteriosus. At the more severe end of this spectrum, involution of the proximal sixth aortic arch leads to absence of the proximal pulmonary artery, with or without continuity of the branch pulmonary arteries.

### Pre-Procedure Planning

**Fig. 2:**
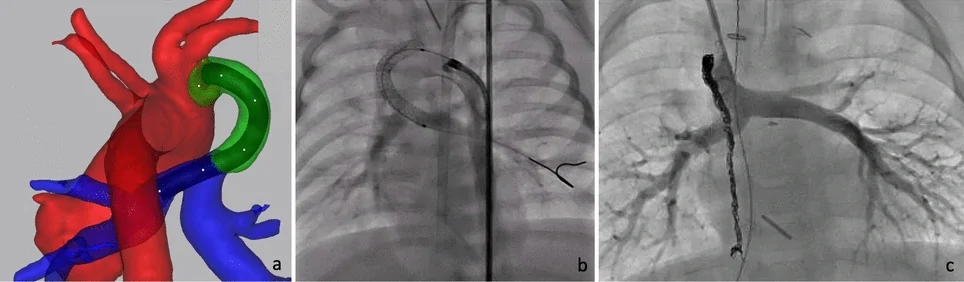
(**a**) 3D CT reconstruction (posterior view) showing the LPA originating from the PDA with a spline used to simulate stent placement. (**b**) Angiogram depicting stent placement into the LPA, jailing the RPA via the umbilical approach. (**c**) Angiogram illustrating the branch pulmonary arteries at the time of the Glenn procedure

Cardiac CT with 3D reconstruction is performed for optimal procedure planning before PDA stent placement. 3D CT reconstructions are created using Mimics Innovation Suite (Materialise, Leuven, Belgium). PDA centerlines are derived, and measurements are taken along both the centerline and a straight end-to-end line, with their ratio indicating tortuosity. Splines (a smooth curve created through a set of points, commonly used in computer graphics for precise and flexible modeling) are created to visualize access and catheter trajectory; splines with lower tortuosity and lower angles at PDA insertion are perceived to lead to nominal access. Splines are also used to approximate the location of the stent. (Fig. 2). [18].

### Surgical Management of the PDA Stent and the Jailed Branch

**Fig. 3:**
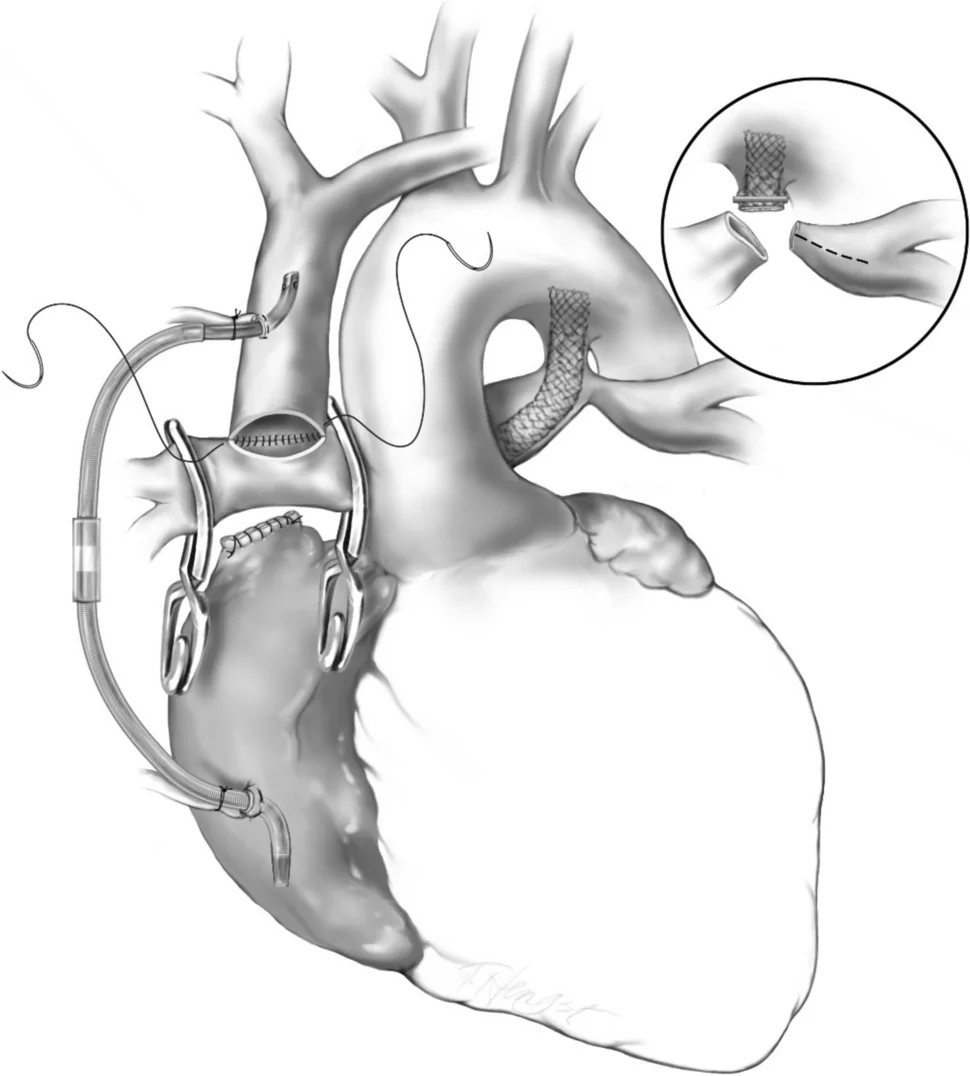
The image illustrates the off-pump technique with a shunt connecting the SVC to the right atrium and bi-directional Glenn construction with running suture. The PDA stent can be seen protruding into the aorta and crossing the ductus into the RPA with a focal point of narrowing between the RPA and LPA representing ductal coarctation. A metal hema-clip is used to occlude the aortic side of the PDA stent as depicted in the inset image. The LPA is then incised to open the vessel to an area of larger caliber and any abnormal (ductal) tissue is excised prior to reconnecting it to the RPA. The back-wall between the RPA and LPA is sewn together primarily and the anterior portion is reconstructed with a pulmonary homograft patch to prevent future narrowing

Patients with branch PAs arising from the PDA are at risk of developing PA discontinuity when the PDA closes (Fig. 1(b)). Pulmonary arteries in this setting typically require reconstruction during their BT shunt surgery or during Glenn/definitive repair. For single ventricle patients, after bidirectional Glenn construction, the PDA stent is isolated from the branch PAs using neuro-clips and occluded with a metal hemaclip at the ductal/aortic origin and removed from the PA. After stent removal, the PA is routinely patched to prevent focal stenosis. Segmental resection is performed for all patients with ductal coarctations with primary back wall reconstruction and anterior homograft patch augmentation. This procedure can be done off-pump in selected patients. Fig. 3

### Analysis

We reviewed images from cardiac catheter angiography, Computed Tomography with and without 3D reconstruction, and echocardiography done before and during initial PDA stent placement as well as before Glenn/definitive surgery. Patients found to have a BPA arising from the PDA with resultant jailing of this branch (BPA jailing), by the protruding stent, were identified. These patients were analyzed for characteristics such as age, weight, and body surface area (BSA) at initial PDA stenting. Cardiac parameters analyzed included diagnosis (cardiovascular anatomy) and pulmonary blood flow source (single vs dual). Patients without antegrade pulmonary blood flow through the pulmonary valve at the time of PDA stenting were considered to have single-source pulmonary blood flow. PDA anatomy was analyzed for its origin and the PDA type was classified as straight (Type I), mildly tortuous/ one turn in ductus (Type II) or severely tortuous/ multiple complex turns in ductus (Type III) [10]. The pulmonary artery size (Nakata index) and symmetry score (diameter ratio of the smaller to larger central BPA) were measured at the time of PDA stenting and prior to Glenn/definitive surgery. Cardiac catheterization technique was analyzed using access site, stent type, and size, stent extension to the main or BPA, and anticoagulation post-PDA stent placement. The primary endpoint was the growth of the jailed pulmonary artery branch, as measured by the Nakata index and symmetry score. The secondary endpoints were complications, reintervention, interstage mortality, and outcomes of patients with BPA jailing.

Data was extracted from patient files accessed through the Electronic Medical Records (EMR) and stored in Redcap. Continuous variables were analyzed using summary statistics such as means (ranges) or median (IQR) and categorical and discrete data were analyzed using percentages/proportions.

## Results

During the study period 22/70 patients (31%) were found to have a BPA arising from the PDA (20 LPA, 2 RPA). As a result, a branch pulmonary artery was inevitably crossed and jailed following ductal stenting. Only those 22 patients were analyzed. The median age at PDA stenting was 9.5 days (range 4–159 days), and the median weight was 3275 g (2600-4200 g). 16/22 (73%) patients had single-source pulmonary blood flow. The PDA tortuosity index varied, with PDA type III being the most common (77%), followed by type II (14%) and type I (9%) (Table 1). The PDA was noted to arise from the underside of the transverse aorta in 18/22 (82%), the right innominate artery in 3/22 (14%), and the left subclavian artery in 1/22 (4%). Procedural techniques are summarized in Table 2. Patients with a PDA arising from the transverse arch were accessed via the axillary (n = 11), femoral artery (n = 1), or carotid (n = 6) approaches, while those with a PDA arising from the subclavian/innominate arteries were accessed through the femoral artery (n = 2) or umbilical artery (n = 2).

**Table 1:** Table showing Patient demographics and cardiovascular anatomy during initial PDA stent placement

N = 22	Median	Range
Demographics		
Age in days	9.5	4–159
Weight in g	3275	2600–4200
BSA in m^2^	0.21	0.18–0.24
	Number	Percent
Cardiovascular anatomy		
Diagnosis		
DORV with PS/PA	7	32%
PA with Atrioventricular canal defect	5	23%
PA/VSD	5	23%
DILV with PA	2	9%
Severe Ebstein anomaly with PA and IVS	2	9%
Tricuspid Atresia/Normally related GA/restricted VSD	1	5%
Branch pulmonary artery arising off the PDA		
Left Pulmonary Artery	20	91%
Right Pulmonary Artery	2	9%
PDA Type		
I	2	9%
II	3	14%
III	17	77%
PDA origin		
Underside of the Aorta	18	82%
Right innominate artery	3	14%
Left subclavian artery	1	5%
Pulmonary blood flow source		
Single	16	73%
Dual	6	27%
Proximal branch pulmonary artery stenosis		
Left Pulmonary Artery	9	41%
Right Pulmonary Artery	1	5%
Bilateral	1	5%

*BSA* Body surface area,* DORV* Double outlet right ventricle,* DILV * Double inlet left ventricle,* PS* Pulmonary stenosis,* PA * Pulmonary atresia,* IVS *Intact ventricular septum,* VSD * Ventricular septal defect,* GA* Great arteries

**Table 2:** Table showing cardiac catheterization technique used during initial PDA stent placement

N = 22	Median	Range
Technique		
Access sites		
PDA arising from transverse arch		
Axillary	11	61%
Carotid	6	33%
Femoral	1	6%
PDA arising from subclavian/innominate arteries		
Femoral	2	50%
Umbilical	2	50%
Branch PA that stent was extended into		
Left Pulmonary Artery	7	32%
Right Pulmonary Artery	9	41%
Main Pulmonary artery	6	27%
Drug-eluting coronary Stent size		
3 mm	2	9%
3.5 mm	19	86%
4 mm	1	5%
Number of stents		
1	17	77%
2	4	18%
3	1	5%
Anticoagulation		
Asprin/plavix	20	91%
Lovenox	2	9%

*PDA* Patent Ductus Arteriosus, *PA* Pulmonary artery

All patients underwent PDA stenting using drug-eluting coronary stents (Frontier Onyx, Medtronic, Minnesota) at 3 mm (n = 2), 3.5 mm (n = 19), and 4 mm (n = 1). 17 patients received one stent, four needed 2 stents, and one needed 3 stents. For patients requiring 1 stent, the median length was 26 mm (range 15–38 mm) while for those needing more than 1 stent, it was 34 mm (range 23–44 mm). Of the 20 patients with the LPA arising from the PDA, nine had the stent extended into the RPA, six into the LPA, and five into the MPA. For the two patients with the RPA arising from the PDA, one had the stent extended into the LPA and the other into the MPA (Table 2).

Two patients required re-intervention with additional stent placement within the same admission due to uncovered ductal tissue -one due to an unrecognized missed pulmonary end and the other due to partial stent migration the next day, resulting in a missed aortic end. There were no procedural or 30-day mortalities. 20/22(91%) were discharged on dual antiplatelet therapy (Aspirin 20.25 mg and Plavix 1 mg/kg/day), while the other 2 were placed on Lovenox due to femoral vein thrombosis, later transitioned to aspirin and Plavix. (Table 2).

### Primary Endpoint

**Fig. 4:**
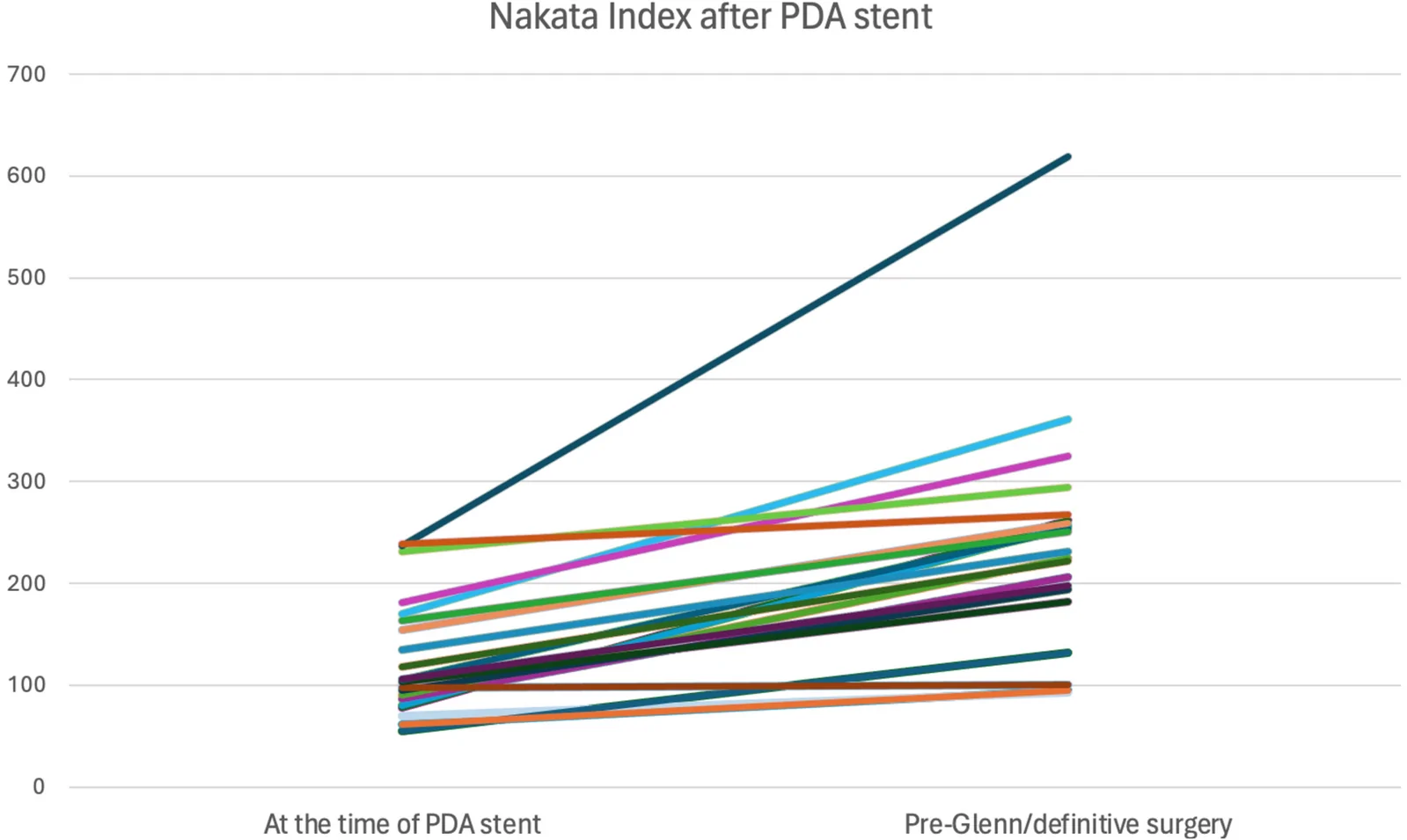
Change in Nakata index from PDA stent placement to pre-Glenn/definitive surgery (N = 21)

BPA measurements were taken by reviewing cardiac angiography images and cardiac CT with and without 3D reconstruction. At the time of PDA stenting, the mean Nakata Index was 129 mm^2^/m^2^ (95% CI [102, 156]) with a median of 106 mm^2^/m^2^ (range 55–237 mm^2^/m^2^). The mean symmetry score was 0.86 (95% CI [0.81, 0.91]), with a median of 0.88 (range 0.57–1). 21/22 patients had BPA measurements taken during Glenn/definitive surgery planning (by angiography during pre-Glenn cardiac catheterization or by CT for cases that had biventricular repair), one patient transferred care to another facility after initial PDA stenting. In these patients, the jailed BPA demonstrated subsequent growth of the distal vessel (Table 3). The mean Nakata Index was 239 mm^2^/m^2^ (95% CI [187, 292]) with a median of 231 mm^2^/m^2^ (range 93–619 mm^2^/m^2^). There was a statistically significant increase in the mean Nakata index by 117.35 mm^2^/m^2^ (95% CI [61.06, 173.64], p-value 0.0001) (Fig. 4). The mean symmetry score decreased from 0.86 to 0.81 (95% CI [0.74, 0.89]), and the median score from 0.88 to 0.86. However, this reduction was not statistically significant (− 0.06, 95% CI [− 0.1479, 0.0279], p-value 0.1751).

**Table 3:**
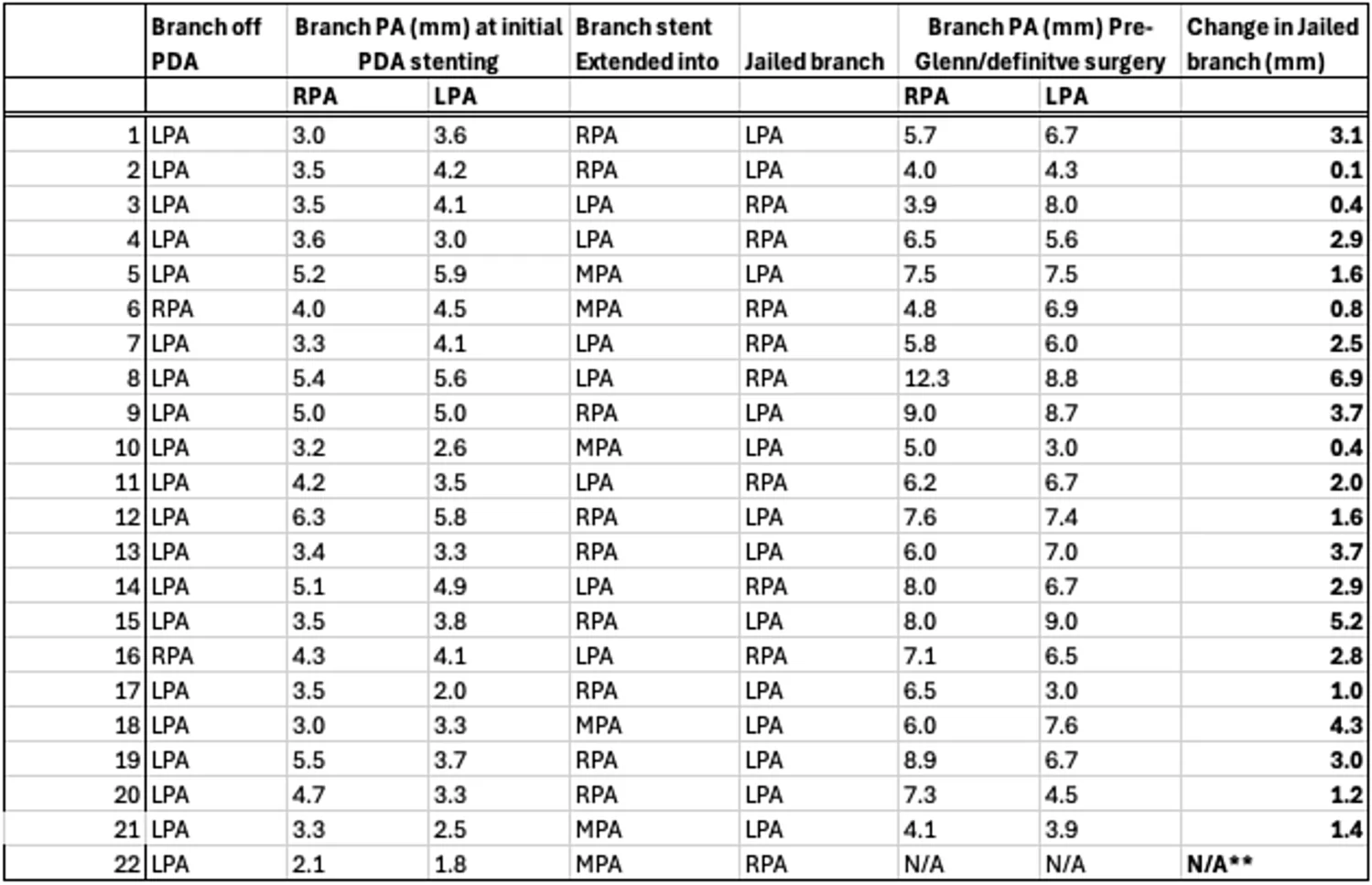
Table demonstrating growth in jailed branch Pulmonary Artery

*PDA* Patent Ductus Arteriosus, *RPA* Right pulmonary artery, *LPA* Left pulmonary artery, *MPA* Main pulmonary Artery

^**^ Patient transferred care to another facility after initial PDA stenting

Flow analysis of individual branch pulmonary arteries (BPAs) was conducted as part of routine pre-Glenn MRI in seven patients. The results showed preferential flow to the jailed BPA in 1 patient (70/30 split), equal flow to both BPAs in 2 patients, and reduced flow in the jailed BPA in 4 patients (30/70, 33/67, 40/60 and 40/60 split respectively). 11/22 patients (50%) had proximal BPA stenosis of the jailed PA branch but still exhibited growth of the distal branch.

Twelve patients progressed to receive a Glenn for single ventricle palliation, five underwent definitive surgical repair, one underwent LPA plasty and BT shunt at 7 months of age, one transferred care to another facility pre-repair, and two are awaiting repair. Two patients in this study cohort died. One was a 24-week premature infant with multiple anomalies who was deemed not a surgical candidate and had a stent placed at 159 days of age. He subsequently underwent balloon dilation of the PDA stent to prolong his palliation and died one year after PDA stenting. The second patient had a perinatal neurologic insult that preceded PDA stenting and died after the Glenn.

**Fig. 5:**
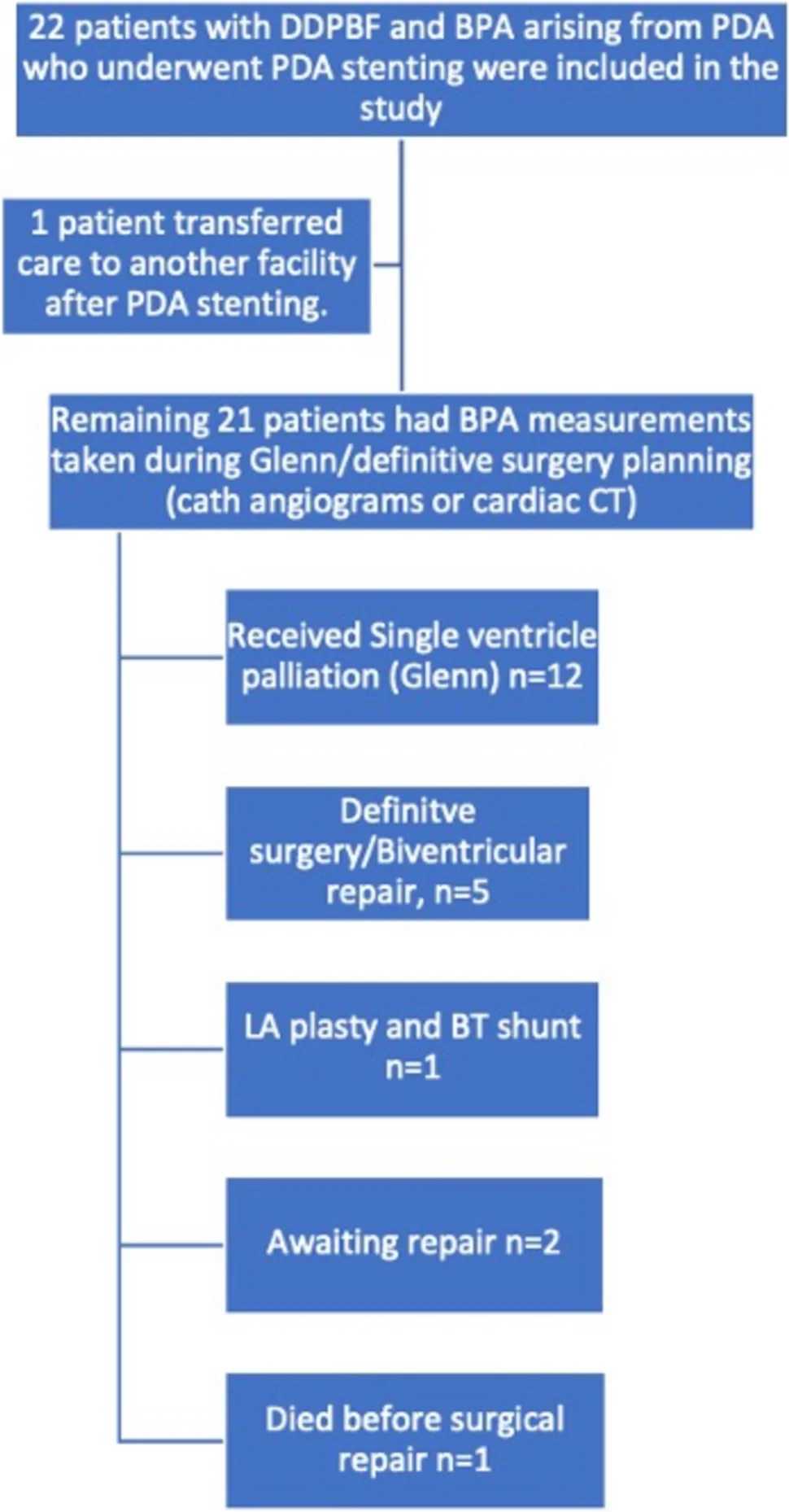
Algorithm showing patients’ journey from initial PDA stenting to Glenn/definitive surgical repair (n = 22)

All eighteen patients (12 Glenn, 5 definitive repairs, 1 BT shunt) also had pulmonary artery plasty during the same surgery (Fig. 3 and Fig 5). Only 12 of the 18 patients had post-Glenn/definitive surgery imaging with BPA measurements, conducted at intervals ranging from 4 to 47 months. There was a non-statistically significant reduction in the mean Nakata index (− 32, 95% CI [− 41, 105], p-value 0.38) and an increase in the mean symmetry score (0.05, 95% CI [− 0.057, 0.1572], p-value 0.3483). However, the median Nakata index was 215 mm^2^/m^2^ (mean 215.9 mm^2^/m^2^), and the median symmetry score was 0.88 (mean 0.85).

**Fig. 6:**
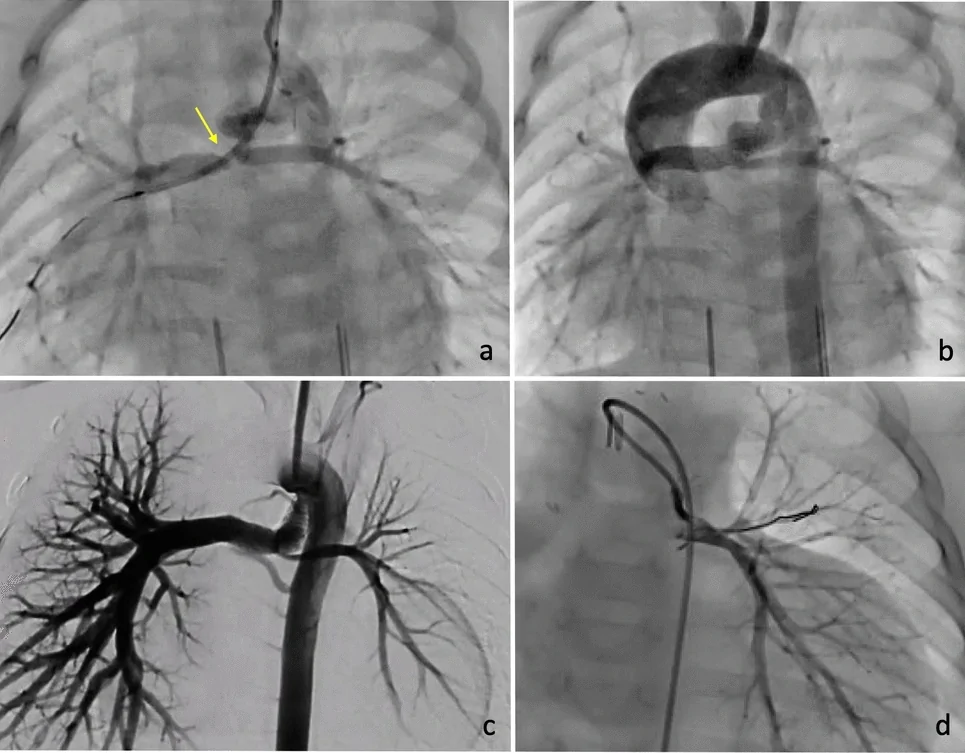
(**a**) Angiogram during PDA stent implantation via the left carotid approach with LPA arising from the PDA. Note hypoplastic LPA and proximal RPA stenosis (arrow). The stent was extended into the RPA jailing the hypoplastic LPA over a stiff wire. (**b**) angiogram after the wire was removed during that same catheterization procedure. (**c**) Angiogram during re-intervention 3 months later. Note interval growth of the RPA, proximal stenosis of the LPA, and no significant growth of the distal hypoplastic LPA. (**d**) Angiogram through the central BT shunt that is directed towards the LPA. RPA is not filling due to the direct engagement of the catheter into the proximal LPA

One patient was noteworthy as they had diffuse LPA hypoplasia (1.7 mm), normal RPA at 3.5 mm with proximal RPA stenosis, Nakata Index of 55, and symmetry score of 0.57 prior to PDA stenting. The stent was extended into the RPA (due to concern about proximal RPA stenosis), thereby jailing the LPA. Strut dilation was not performed during that procedure as per our usual protocol due to concern regarding manipulation through a freshly placed stent. They required interval ballooning of the PDA stent and LPA through the struts due to hypoxia. At the Pre-Glenn cardiac catheterization done at 7 months of age, the RPA was found to have grown significantly to 6.5 mm, while the LPA had suboptimal growth to 3 mm. This patient proceeded to have pulmonary artery plasty and a BT shunt to encourage LPA growth (Fig. 6).

### Secondary Endpoints

**Fig. 7:**
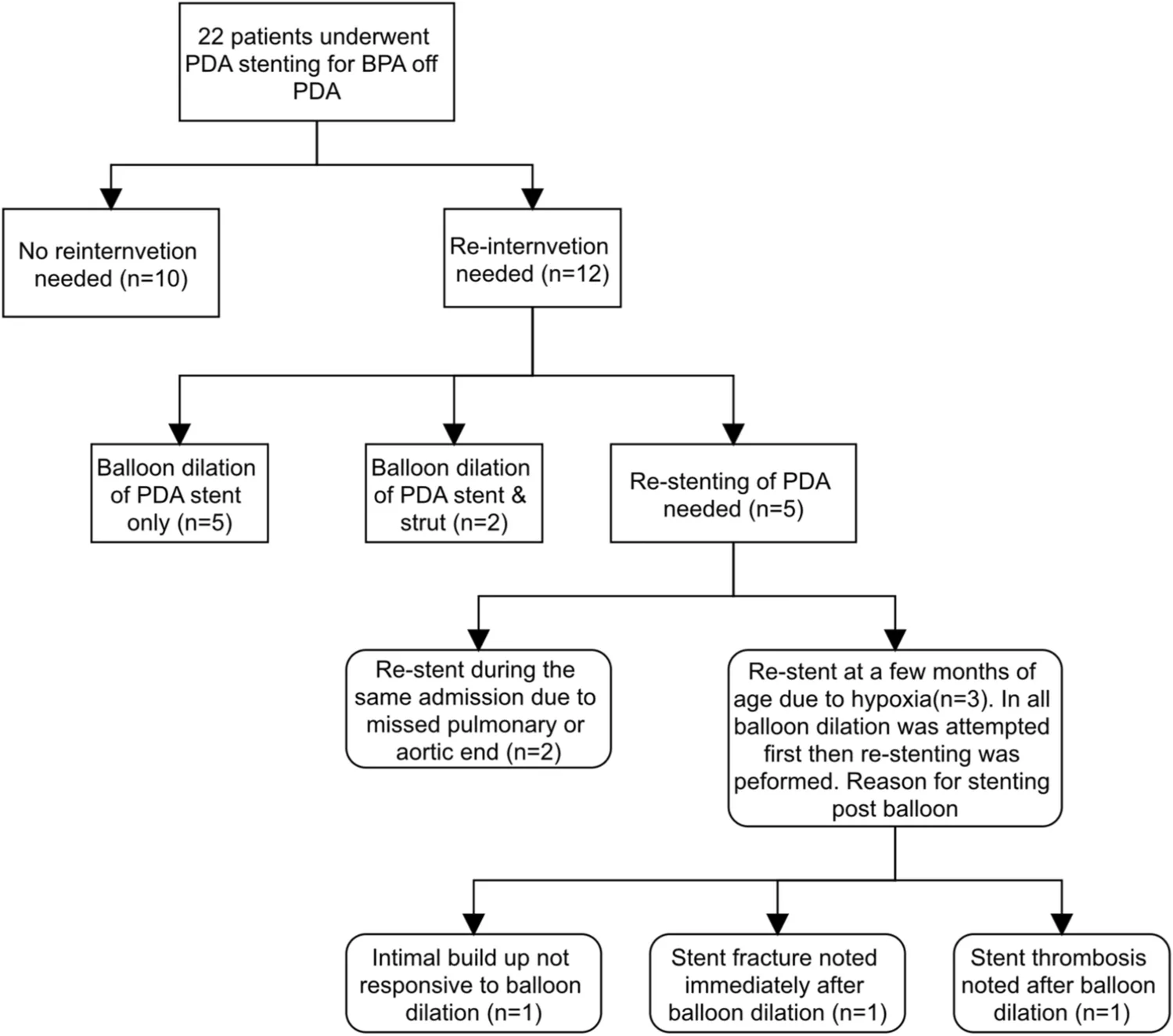
Re-interventions done for hypoxia between initial PDA stenting and Glenn/definitive surgical repair

Re-intervention for hypoxia was required in 12 out of 22 patients (55%) (Fig. 7). Among these:

- PDA stent ballooning alone: Performed in 5 patients.
- Balloon dilation of PDA stent as well as Strut dilation for jailed branch flow: 2 patients underwent ballooning of the PDA stent as well as stent strut dilation to address concerns about inadequate blood flow to the jailed branch pulmonary artery.
- Re-stenting: Required in 5 patients:
  - Two patients were re-stented during the same admission as the initial PDA stenting due to missed pulmonary or aortic end.
  - Two patients developed progressive hypoxia a few months later. In one case, initial PDA stent balloon dilation failed due to intimal build-up, necessitating PDA re-stenting. In the other, stent fracture immediately following balloon dilation required re-stenting.
  - One patient experienced stent thrombosis a few hours after balloon dilation, requiring emergent stent placement during a return to the catheterization laboratory.

Notably, one patient had a stent fracture at the left pulmonary artery (LPA) take-off and was referred for early Glenn surgery (Fig. 5).

## Discussion

Transcatheter PDA stenting has recently gained acceptance as the preferred primary procedure to secure pulmonary blood flow in infants born with duct-dependent pulmonary circulation. In 2018, RCHSD adopted PDA stenting as the primary procedure to establish pulmonary blood flow for neonates with duct-dependent pulmonary circulation who could not undergo primary repair. BPA jailing, however, remains a significant potential scenario in PDA stenting and is associated with adverse effects including restricted growth of the jailed branch, hypoxia, stent thrombosis, and unilateral pulmonary edema or hemorrhage. Authors like Qureshi et al. have found that BPA jailing incidence is higher in patients with higher PDA tortuosity index [10]. Similarly, Aggarwal et found that BPA jailing in patients with and without preexisting proximal branch PA stenosis was more common in cases with higher PDA tortuosity and was also associated with higher reintervention rates. Both authors however found that BPA jailing does not result in significant restriction in BPA growth or symmetry [10, 13]. These and other similar findings have influenced current practice such that factors like PDA tortuosity, proximal BPA stenosis, and small pulmonary artery size are no longer considered contraindications to PDA stenting.

It is important to distinguish between the BPA inadvertently jailed by the PDA stent and the BPA arising from the PDA (Fig. 1). While both scenarios involve crossing of a BPA by the stent, the latter by definition involves the stent crossing and jailing that pulmonary artery branch. We present 22 patients with BPA jailing in this later scenario. It is unclear from previous studies whether there is a predilection for the right or left BPA to arise from the PDA. Our study however found that an overwhelming majority of patients (20/22) had the left pulmonary artery arising from the PDA.

BPAs arising from the PDA are notably prevalent in conditions where fetal circulation experiences an early gestational lack of forward flow, prompting an early reversal of the pulmonary artery flow such as pulmonary atresia with a ventricular septal defect (VSD) [10]. These cases typically involve PDAs originating from the underside of the aortic arch, a finding that was also observed in our study. Despite the wide variety of cardiac diagnoses in our study, all were associated with pulmonary atresia/severe pulmonary stenosis and interventricular communication. Additionally, 82% of the patients had PDAs arising from the underside of the aorta. This distinction stands in stark contrast to situations characterized by PDA-dependent pulmonary blood flow, wherein diminished pulmonary artery flow manifests later in gestation, as observed in conditions like pulmonary atresia with an intact ventricular septum or tricuspid atresia. Our observations in these diagnoses consistently revealed continuous branch pulmonary arteries, indicative of their non-origin from the PDA (Fig. 1 (a)).

Given that PDA stenting is still in the early stages of adoption, there is no consensus on which BPA the stent should be extended into when a BPA arises from the PDA. One approach is to extend the stent into the BPA that originates from the PDA, ensuring complete coverage of ductal tissue to prevent the branch from becoming isolated after PDA closure. Another approach is to stent into the contralateral BPA, as the inter-segment between the branch pulmonary arteries can be seen as a continuation of the PDA and may also contain ductal tissue that could compromise the vessel if left uncovered (Fig. 2). Additionally, it is hypothesized that extending the stent into a BPA with proximal stenosis could encourage distal growth. Despite these strategies, it is still expected that most patients will require pulmonary artery plasty during Glenn or definitive surgery.

Our institutional practice has evolved to include cardiac CT with 3D reconstruction for optimal procedure planning before PDA stent placement [18]. We preferentially plan to stent into the branch contralateral to the BPA arising from the PDA (Fig. 2). In cases of proximal BPA stenosis, we prioritize stenting into the BPA with the proximal stenosis. However, the final placement of the PDA stent also depends on the path the guide wire takes. Ideally, it passes without significant resistance or manipulation to avoid PDA dissection or thrombosis which can occur if there is rough handling of the PDA during catheter and wire manipulation.

For the previously mentioned patient with a hypoplastic LPA and proximal RPA stenosis (Fig. 5), the PDA stent was extended into the RPA with suboptimal LPA growth. In retrospect, it could be argued that extending the stent into the hypoplastic LPA might have been a more favorable approach. However, it was difficult to predict how the RPA would respond with the uncovered inter-segment PDA tissue constricting. Additionally, authors like Koneti et al. advocate for strut dilation following ductal stenting in cases with proximal BPA stenosis to augment pulmonary blood flow [19]. However, at our institution, we do not routinely perform strut dilation during initial ductal stenting, as the PDA has not yet conformed to the stent. We feel that extra manipulation at this stage could result in PDA dissection or thrombosis and may not be necessary if adequate flow is seen into that branch. Ultimately, close surveillance of the jailed branch is warranted in patients with similar anatomy, with early re-intervention if needed. In such cases, early surgical pulmonary artery plasty and a BT shunt should be considered.

All except one of the jailed BPAs showed statistically significant growth. The symmetry score is an important measure as it assesses differential growth of the branch pulmonary arteries. Of the twenty patients in our cohort who had pre-Glenn/definitive surgery measurements of their branch pulmonary arteries, twelve had a reduction in their symmetry score while eight had an increase. The change in symmetry score ranged from − 0.37 to 0.25, which may indicate variable BPA growth rates influenced by factors such as restricted blood flow, over-circulation to one BPA, or post-stenotic dilation. This change was, however, not statistically significant which reflects that PDA stenting in these cases did not disrupt the branch PA symmetry.

The long-term success of single ventricle palliation is dependent on having good-caliber pulmonary arteries. Therefore, the initial palliative procedure to establish pulmonary blood flow should facilitate adequate growth of the pulmonary arteries. Previous reports have shown that pulmonary artery growth in patients who have undergone PDA stenting is comparable to those who have been palliated with BT shunts [1–4]. We have shown a significant increase in the Nakata index between the time of PDA stenting and catheterization/CT done prior to Glenn/definitive repair with no statistically significant change in the symmetry score. Half (11 out of 22) of our patients with proximal BPA stenosis of the jailed branch post stenting, exhibited good growth in the distal branch (a form of post stenotic dilation pattern). All 17 patients who underwent Glenn or definitive repair required pulmonary artery plasty at the time of PDA stent removal. It could be argued, however, that patients with a BPA originating from the PDA would likely require pulmonary artery plasty even if a BT shunt had been placed.

For the twelve patients that had imaging post-Glenn/definitive repair, the median Nakata index was 215 mm^2^/m^2^ (mean 215.9 mm^2^/m^2^). The non-statistically significant reduction in the mean Nakata index likely exhibits the previously reported slight decrease in the Nakata index after conversion to a non-pulsatile circulation with a Glenn [14]. It is however difficult to draw firm conclusions from these findings given the limited dataset.

Previous studies have shown that PDA stenting is associated with higher reintervention rates when compared to BT shunts [1, 14]. Our study had similar findings with a re-intervention rate of 55% which included ballooning, re-stenting and strut dilation. Notably, we experienced one case of thrombosis, which occurred during re-intervention and was not detected until after the patient had left the catheterization lab. None of our patients developed thrombosis during the initial stent placement. Thrombosis typically arises during dilation of a pre-existing stent, particularly if intimal enfolding has occurred. We believe that dilation may disrupt the intimal layer leading to increased risk of thrombosis, and we are prepared to immediately re-stent. We have become more vigilant in preventing this complication, particularly during re-intervention. To mitigate this risk, we ensure adequate heparinization with 100 u/kg at the start of the procedure and rigorously monitor activated clotting time (ACT) levels every 30 min to maintain it above 250 throughout the case. Additionally, we administer a Plavix reloading dose (2 mg/kg) in the catheterization lab following dilation or re-stenting before the patient leaves the room.

## Ethical Considerations

Ethical approval to conduct this study was obtained from the University of California, San Diego IRB. Patient confidentiality was maintained throughout the study by encrypting personal information. Access to the data was limited to the primary investigator and research assistants. Consent from the study subjects was not required, as the data was extracted retrospectively from electronic medical records.

## Study Limitations

This retrospective study reflects the practice and outcomes of a single center, which may be influenced by unique institutional preferences and the experience and skill of the providers involved. As such, the findings may not be universally applicable. Additionally, the long-term outcomes of these patients have not yet been established. The small sample size of this study reduces its statistical power, though the statistically significant findings are consistent with those of larger studies.

We acknowledge the concern regarding long-term radiation exposure from including pre-procedure cardiac CT in our management approach. However, our CT protocols are optimized to minimize radiation exposure. Additionally, we believe the pre-procedure planning benefits obtained from cardiac CT with 3D reconstruction outweigh the risks.

## Conclusion

PDA stenting is a feasible option for patients with a branch pulmonary artery arising from the PDA. While the procedure is associated with a relatively high rate of re-intervention, it has been demonstrated to support adequate growth of the distal BPA and to maintain pulmonary artery symmetry. Regular monitoring of the jailed BPA is recommended, and further studies are needed to validate these early findings.

## Data Availability

No datasets were generated or analysed during the current study.
